# Upper Cervical Tuberculosis in a Young Child

**DOI:** 10.4269/ajtmh.23-0226

**Published:** 2023-08-07

**Authors:** Shutao Gao, Fulati Mamat, Weibin Sheng

**Affiliations:** Department of Spine Surgery, Xinjiang Medical University Affiliated First Hospital, Urumqi, China

**Figure 1.**
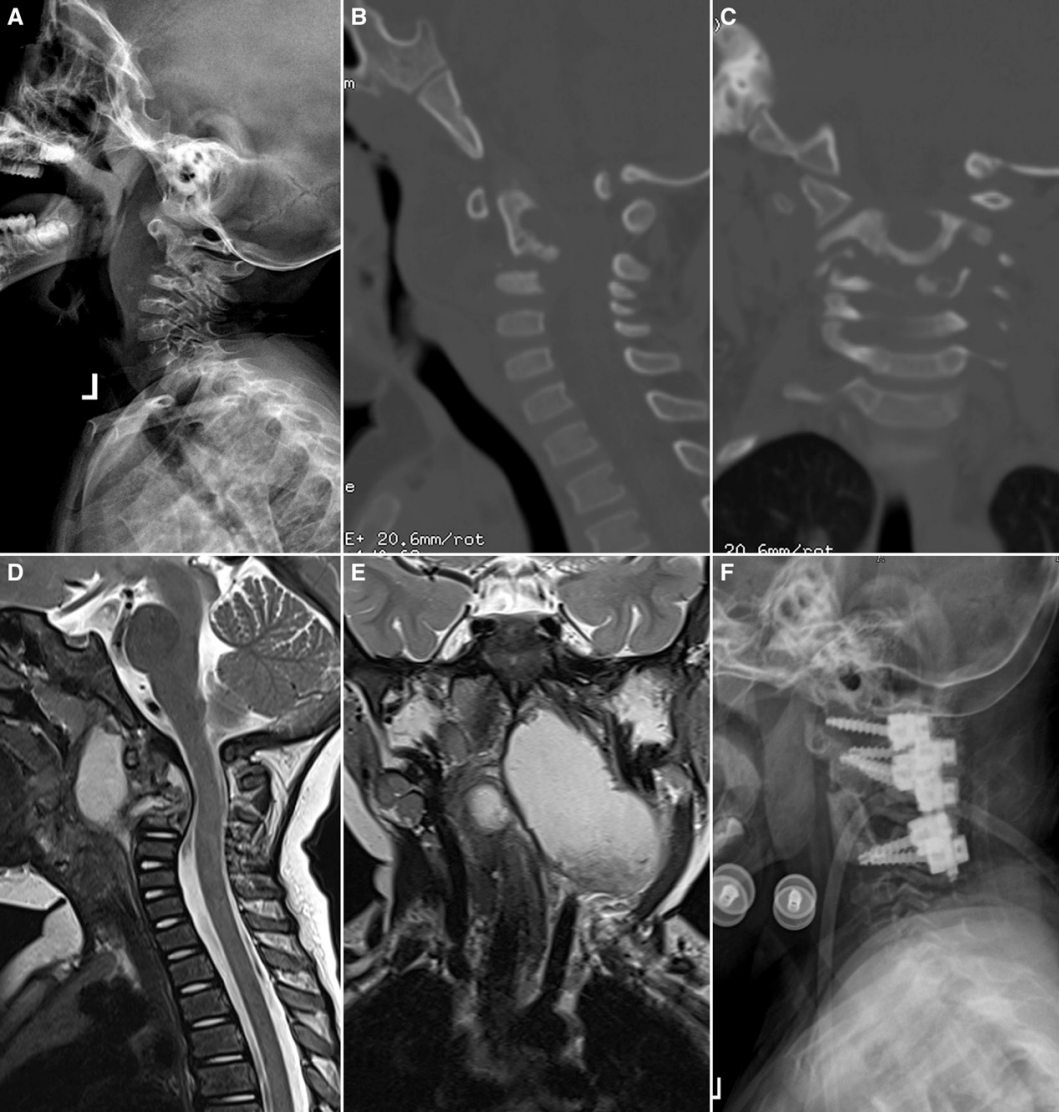
(**A**) Preoperative cervical plain radiograph. (**B** and **C**) Computed tomography scans indicated bone destruction of C2 and C3 vertebrae. (**D** and **E**) Magnetic resonance imaging showed a massive paraspinal abscess. (**F**) Postoperative cervical plain radiograph.

A 3-year-old female child was brought to the outpatient department with 1.5 months of progressive gait difficulty and neck pain. Physical examination showed grade 3/5 strength of the upper and lower extremities and hyperactive deep tendon reflexes. Laboratory tests exhibited a normal white cell count, an increased erythrocyte sedimentation rate (ESR; 97 mm/hour), an elevated C-reactive protein level (CRP; 130 mg/L), and a positive interferon-gamma release assay (T-spot test). Lateral cervical plain radiograph showed an abnormal cervical alignment (Figure 1A). Computed tomography scans indicated bone destruction of C2 and C3 vertebrae (Figure 1B and C). Magnetic resonance imaging showed a massive paraspinal abscess (Figure 1D and E).

**Figure 2.**
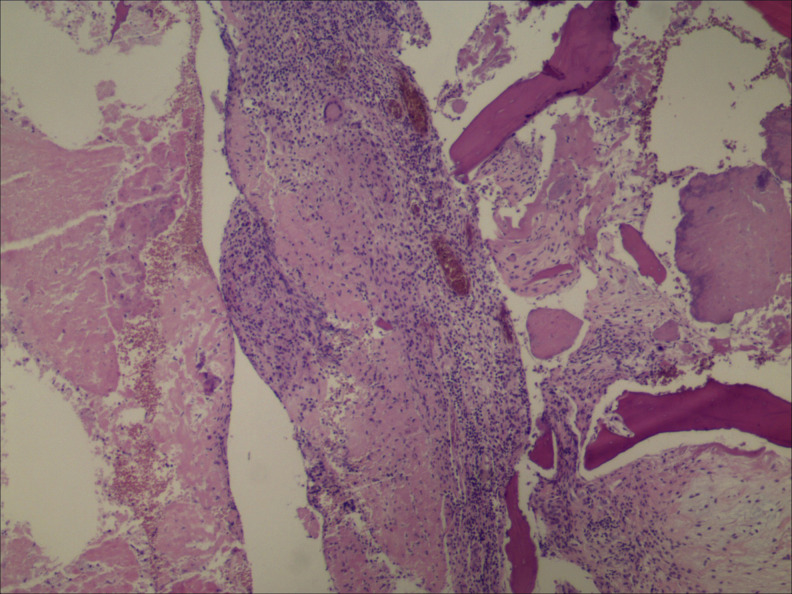
Histopathology showed extensive granulomata mixed with caseous necrosis (40×).

The imaging examinations and laboratory tests supported the diagnosis of cervical tuberculosis (TB). The child underwent surgical treatment with one-stage posterior internal fixation followed by anterior-approach debridement and bone grafting. Histopathological examination showed extensive granulomata mixed with caseous necrosis. The child’s symptoms significantly improved after surgery (Figure 1F). The histopathological findings indicated extensive granulomata mixed with caseous necrosis, supporting the diagnosis of TB (Figure 2). Therefore, anti-TB chemotherapy (rifampicin 10 mg/kg/day, isoniazid 10 mg/kg/day, pyrazinamide 25 mg/kg/day, ethambutol 15 mg/day) was prescribed. At 5-month follow-up, the child’s strength recovered to grade 5/5, and the ESR and CRP values returned to normal.

Upper cervical TB in young children is rarely reported. Without timely and proper treatment, affected individuals may suffer from progressive neurological deficits and disability.^1^ Although anti-TB chemotherapy is the mainstay of treatment, surgical treatment is necessary for patients with progressive kyphotic deformity, neurological deficit, and a large abscess.^2^ Because young children have great spinal growth potential, frequent follow-ups are encouraged.
